# Effect of Beta-Blocker Consumption on the Severity and Extension of Perfusion Defects in Dipyridamole Myocardial Perfusion Single-Photon Emission Computed Tomography

**DOI:** 10.1055/s-0044-1787887

**Published:** 2024-06-24

**Authors:** Shirin Shahlaee, Homa Falsoleiman, Mahdi Hasanzade Daloee, Arash Gholoobi, Ghasem Ali Divband, Nasrin Raeisi, Vahid Reza Dabbagh Kakhki

**Affiliations:** 1 Nuclear Medicine Research Center, Mashhad University of Medical Sciences (MUMS), Mashhad, Iran; 2 Cardiovascular Department, Mashhad University of Medical Sciences, Mashhad, IRAN

**Keywords:** CAD, SPECT, beta-blockers, dipyridamole, myocardial perfusion

## Abstract

**Background** Regarding the less-known effects of beta-blocker consumption on the diagnostic value of the myocardial perfusion scan with dipyridamole stress in coronary artery disease (CAD), we aimed to compare the findings of the scans done on the beta-blocker consumption course and after discontinuation of this medications.

**Materials and Methods** Thirty patients with probably CAD and abnormal myocardial perfusion scans (presence of reversible defect), who had been treated with beta-blockers for at least 3 months, were studied. Dipyridamole stress phase of myocardial perfusion single-photon emission computed tomography (SPECT) was performed two times with an interval of about 1 week, once after discontinuation of all antianginal and anti-ischemic medications, statins, and beta-blockers for 72 hours prior to the study, and again after discontinuation of all these medications except for beta-blockers. Imaging was done with the same protocol, radiopharmaceutical dose, and imaging parameters. Summed stress score (SSS), summed stress rest, and summed difference scores (SDS), total perfusion deficit (TPD), severity, and extension of myocardial perfusion defects in three coronary artery territories were analyzed, using quantitative perfusion SPECT software.

**Results** Most variables such as SSS, SDS, TPD, severity, and extension of defects showed a significant difference between the two conditions including beta-blocker consumption and after discontinuing beta-blocker consumption before stress imaging ( *p* < 0.05). Moreover, in patients on treatment with metoprolol, all studied factors including SSS, SDS, TPD, severity, and extension of perfusion defects were significantly reduced when patients consumed beta-blockers before SPECT evaluation ( *p* < 0.05).

**Conclusion** Beta-blocker consumption can lead to a decrease in the severity and extent of myocardial perfusion defects and therefore probably a decrease in the sensitivity of myocardial scans. Discontinuation of beta-blocker prior to the dipyridamole myocardial perfusion scan can improve diagnostic accuracy.

## Introduction

Coronary artery disease (CAD) is a disorder that occurs due to atherosclerotic occlusions of the coronary arteries and is manifested by stable or unstable angina, myocardial infarction, or sudden cardiac death. 1 CAD is the leading cause of cardiovascular mortality in the world. 2 Therefore, appropriate diagnostic methods are necessary for diagnosis and prognostication in CAD.

Myocardial perfusion single-photon emission computed tomography (MSPECT) is a diagnostic method that measures blood flow in the myocardium and makes it possible to diagnose myocardial ischemia or infractions. 3 MSPECT provides valuable information about the severity and extension of CAD, which effects the patient's risk, prognosis, and better management. 4 This method is often examined separately in two phases: stress (pharmacological or exercise stress) and rest phases. 5 Pharmacologic stress agents include vasodilators such as adenosine and dipyridamole as well as inotropic-chronotropic agents such as dobutamine. 6 Dipyridamole exerts its pharmacological effects by blocking the cellular uptake of adenosine and so increasing the blood levels of endogenous adenosine. 7

In recent years, much research has been done to increase the diagnostic accuracy of myocardial perfusion scan. 8 9 10 One of the most challenging factors affecting the diagnostic accuracy of dipyridamole myocardial perfusion scan is the cessation or continuation of beta-blockers consumption before the stress phase. Beta-blockers are one of the most important medications used in the treatment of CAD that block beta-adrenergic receptors in the target cells 11 and cause to decrease cardiac output, stroke volume, and arterial blood pressure by reducing heart rate and myocardial contractility. 12 Thus, discontinuation of beta-blockers in treated patients can increase the risk of ischemic attack or complications such as sudden rise of blood pressure and heart rate. Some studies suggest that holding of beta-blockers is not mandatory before diagnostic scan, and, on the other hand, others have suggested that beta-blockers may reduce the presence and severity of cardiac perfusion defects in postdipyridamole stress images. 13 14 15 So, the effect of beta-blockers on the diagnostic value of MSPECT and the severity of dipyridamole-induced perfusion defects is controversial.

Uncertainty and limited studies about the effect of beta-blockers on MSPECT results led us to investigate the impact of beta-blockers on the dipyridamole stress /rest gated myocardial perfusion SPECT variables.

## Materials and Methods

### Selection of Patients

Thirty patients with abnormal MSPECT (23 patients with ischemia and 7 patients with ischemia + infarction) were studied. After obtaining informed written consent, patient underwent dipyridamole stress myocardial SPECT, two times on 2 separate days. First stress imaging was performed after holding beta-blockers for 72 hours prior to the scan, and the second stress imaging was done on the beta-blocker consumption, 1 week later. In both stress imaging, other antianginal and anti-ischemic medications and statins were discontinued for 72 hours. Inclusion criteria were patients with ischemic heart disease and presence of reversible defects in their myocardial perfusion scans who were treated with beta-blockers for at least 3 months. Exclusion criteria were shorter course of treatment, incorrect medicine usage, and patient's dissatisfaction to enter the study.

### Dipyridamole Stress and Single-Photon Emission Computed Tomography

Pharmacological stress was performed with infusion of 0.56 mg/kg dipyridamole over 4 minutes and radiopharmaceutical (740–925 megabecquerel of [ ^99m^ Tc]Tc-Sestamibi) injected 4 minutes after the infusion of dipyridamole. During dipyridamole infusion, heart rate, blood pressure, electrocardiogram, and patients' symptoms were monitored continuously.

One hour after the injection of radiopharmaceutical, imaging was done by the gated SPECT method, Siemens gamma camera and GE (dual head variable angle), E-soft software, and low-energy high-resolution collimator in the supine position. Imaging was acquired in 32 projections (20 seconds each projection) from right anterior oblique to left posterior oblique using matrix: 64 * 64 and 140 keV photopeak (15% energy window) and 1.45 magnification. In gated SPECT imaging, eight frames per cycle and 20% window were used. Then, images were reconstructed with filtered back projection and Butterworth filter (order: 5 and cutoff frequency: 0.55).

One week after the first round of pharmacological stress, stress testing was performed again with dipyridamole, without discontinuation of beta-blockers (carvedilol, metoprolol, propranolol, and atenolol) but with discontinuation of other antianginal, anti-ischemic medications, and statins for 72 hours prior the scan. Imaging protocol, radiopharmaceutical dose, injection-imaging time interval, and all imaging parameters were the same between the two stress phases. The stress and rest phases images were interpreted separately by two experienced nuclear medicine specialists in the field of nuclear cardiology using 17-segment method. Interpreters were unaware about the beta-blocker usage status of the patients.

Using quantitative perfusion SPECT (QPS) software, perfusion parameters such as summed stress score (SSS), summed rest score, summed difference score (SDS), and total perfusion deficit (TPD) were recorded. Also using severity and extension polar maps, 16 17 stress severity score, reversibility severity score, stress extension score, and reversibility extension score in left anterior descending (LAD), left circumflex (LCX), and right coronary artery (RCA) territories were analyzed.

Finally, by analyzing and comparing the obtained data, the effect of beta-blockers on the diagnostic accuracy of myocardial perfusion scan with dipyridamole was investigated.

## Statistical Analysis

Frequency descriptive methods will be discussed, including frequency distribution tables, graphs, central indices, and appropriate scatter to describe the studied variables. Kruskal–Wallis test was used to determine the correlation between myocardial perfusion scan indices after discontinuation and after beta-blocker. The chi-square test was used to determine the relationship between qualitative variables between patients. The significance *p* -value was considered < 0.05, and all the statistical analyses were processed by SPSS software (statistical package, version 24).

## Results

In our study, 43.3% of subjects were male and 56.7% were female. The mean age of participants was 59.27 ± 10.16 years old. Moreover, seven patients had ischemia with infarction (23.4%) and 23 patients only had ischemia (76.6%). Patient's characteristics are demonstrated in Table 1 .

**Table 1:** Patient's characteristics

Variable		Value
Age		59.27 ± 10.16 y old
Sex	Men	13 (43.3%)
	Women	17 (56.7%)
CABG surgery		9 (30%)
Hyperlipidemia		12 (40%)
Diabetes mellitus		14 (46.7%)
Hypertension		24 (80%)
Abnormal findings in angiography		12 (40%)
Type of beta blocker	Metoprolol	25 (83.3%)
	Carvedilol	5 (16.7%)
Scan report	Ischemia and infarction	7 (23.4%)
	Ischemia	23 (76.6%)

Abbreviation: CABG, coronary artery bypass grafting.

Most indices including SSS, SDS, TPD, severity, and extension of perfusion defects in RCA, LCX, and LAD territories were significantly different between the two studied conditions including continuation and discontinuation of beta-blocker consumption before SPECT scan ( *p* < 0.05). However, the severity reversibility score in the LAD territory was the only index that did not differ significantly between the two situations ( *p* = 0.07). The results are shown in Table 2 .

**Table 2:** Myocardial perfusion scan indices in beta-blocker consumer group in two situations including on consumption and after discontinuation of beta-blocker

Variable	After beta-blocker consumption	After beta-blocker discontinuation	*p* -Value
Summed stress score	5.62 ± 6.03	4.12 ± 8.20	0.0001
Summed difference score	4.67 ± 4.07	2.89 ± 6.23	0.0001
Summed rest score	1.97 ± 2.09	1.97 ± 2.09	–
Severity LAD stress	2.33 ± 1.56	2.24 ± 1.63	0.0001
Severity LCX stress	2.71 ± 1.42	2.65 ± 1.69	0.0001
Severity RCA stress	0.33 ± 0.52	0.42 ± 0.33	0.0001
Severity LAD reversibility	0.72 ± 0.78	0.45 ± 0.89	0.07
Severity LCX reversibility	0.64 ± 0.88	0.75 ± 0.9	0.0001
Severity RCA reversibility	0.48 ± 0.71	5.77 ± 2.16	0.001
Extension LAD stress	14.72 ± 10.17	13.62 ± 15.13	0.0001
Extension LCX stress	22.23 ± 9.80	23.53 ± 16.40	0.0001
Extension RCA stress	8.81 ± 3.03	5.68 ± 2.17	0.0001
Extension LAD reversibility	12.35 ± 6.10	10.41 ± 10.86	0.01
Extension LCX reversibility	9.54 ± 4.71	14.17 ± 10.53	0.001
Extension RCA reversibility	9.44 ± 3.03	5.77 ± 2.16	0.0001
Total perfusion deficit LAD stress	5.48 ± 4.25	4.53 ± 5.40	0.0001
Total perfusion deficit LCX stress	3.96 ± 1.96	3.85 ± 2.94	0.0001
Total perfusion deficit RCA stress	1.30 ± 0.52	1.06 ± 0.42	0.0001
Total perfusion deficit (total stress)	8.56 ± 7.48	7.38 ± 10	0.0001
Ejection fraction stress	67.20 ± 14.62	66.57 ± 14.34	0.0001

Abbreviations: LAD, left anterior descending; LCX, left circumflex; RCA, right coronary artery.

In the group of patients who only used metoprolol, all studied indices including SSS, SDS, TPD severity, and extension scores were significantly reduced when patients continued beta-blocker consumption before the stress phase of the study ( *p* < 0.05) ( Table 3 ).

**Table 3:** Myocardial perfusion scan indices in the metoprolol consumer group in two situations including on consumption and after discontinuation of metoprolol

Variable	After metoprolol consumption	After metoprolol discontinuation	*p* -Value
Summed stress score	5.20 ± 5.13	7.88 ± 3.78	0.0001
Summed difference score	3.16 ± 3.81	5.48 ± 2.11	0.0001
Summed rest score	2.04 ± 2.24	2.04 ± 2.24	–
Severity LAD stress	1.60 ± 2.55	1.69 ± 2.45	0.0001
Severity LCX stress	1.28 ± 2.55	1.58 ± 2.56	0.0001
Severity RCA stress	0.23 ± 0.30	0.27 ± 0.33	0.001
Severity LAD reversibility	0.73 ± 0.76	0.84 ± 0.44	0.17
Severity LCX reversibility	0.048 ± 0.48	0.60 ± 0.61	0.01
Severity RCA reversibility	0.67 ± 0.49	1.03 ± 5.04	0.006
Extension LAD stress	9.28 ± 15.03	15.36 ± 14.29	0.001
Extension LCX stress	7.12 ± 15.56	14.00 ± 18.24	0.0001
Extension RCA stress	1.40 ± 3.77	1.36 ± 4.85	0.0001
Extension LAD reversibility	4.80 ± 12.07	10.64 ± 11.01	0.03
Extension LCX reversibility	3.37 ± 7.07	9.20 ± 12.06	0.001
Extension RCA reversibility	3.96 ± 1.24	5.04 ± 1.36	0.0001
Total perfusion deficit LAD stress	3.96 ± 5.74	5.36 ± 4.69	0.0001
Total perfusion deficit LCX stress	1.45 ± 2.82	2.58 ± 3.02	0.0001
Total perfusion deficit RCA stress	0.28 ± 0.60	0.27 ± 0.86	0.0001
Total perfusion deficit (total stress)	6.56 ± 7.38	9.20 ± 6.76	0.002

Abbreviations: LAD, left anterior descending; LCX, left circumflex; RCA, right coronary artery.

Evaluating the carvedilol consumer group (five patients) demonstrated that SSS, SDS, as well as stress severity score, stress extension score, and TPD in the stress phase in RCA and LCX territories as well as extension reversibility score in RCA territory were significantly increased after carvedilol consumption compared to carvedilol withdrawal phase ( Table 4 ).

**Table 4:** Myocardial perfusion scan indices in the carvedilol consumer group, in two situations including on consumption and after discontinuation of carvedilol

Variable	After carvedilol consumption	After carvedilol discontinuation	*p* -Value
Summed stress score	10.20 ± 6.72	9.80 ± 5.76	0.0001
Summed difference score	8.60 ± 6.34	8.20 ± 5.31	0.0001
Summed rest score	1.60 ± 1.14	1.60 ± 1.14	–
Severity LAD stress	1.34 ± 0.49	1.32 ± 0.55	0.17
Severity LCX stress	2.16 ± 3.34	2.24 ± 3.35	0.002
Severity RCA stress	0.82 ± 1.04	0.66 ± 0.68	0.002
Severity LAD reversibility	1.06 ± 0.43	1.16 ± 0.43	0.31
Severity LCX reversibility	1.44 ± 1.80	1.50 ± 1.67	0.03
Severity RCA reversibility	0.92 ± 0.46	6.02 ± 8.01	0.1
Extension LAD stress	14.60 ± 13.61	14.00 ± 11.31	0.2
Extension LCX stress	43.14 ± 23.20	42.47 ± 28.40	0.01
Extension RCA stress	11.20 ± 19.48	6.20 ± 8.25	0.01
Extension LAD reversibility	12.60 ± 12.93	12.00 ± 7.48	0.38
Extension LCX reversibility	11.40 ± 17.09	17.20 ± 20.70	0.44
Extension RCA reversibility	12.00 ± 20.78	6.20 ± 8.01	0.01
Total perfusion deficit LAD stress	5.66 ± 4.15	5.74 ± 4.11	0.12
Total perfusion deficit LCX stress	4.50 ± 7.50	4.72 ± 6.91	0.01
Total perfusion deficit RCA stress	1.70 ± 2.82	1.18 ± 1.69	0.001
Total perfusion deficit (total stress)	14.20 ± 11.98	14.00 ± 9.82	0.07

Abbreviations: LAD, left anterior descending; LCX, left circumflex; RCA, right coronary artery.

## Discussion

MSPECT introduced as a valuable diagnostic and prognostic method for patients with CAD and stress intervention by vasodilator agents is a main component of the study to identify recognizable myocardial defects. 18 19 20 21

Given the controversial findings about the impact of cardiac medications especially beta-blockers on the diagnostic value of MSPECT, we investigate the effect of beta-blocker agents on diagnostic indices of dipyridamole myocardial perfusion scan.

**Fig. 1:**
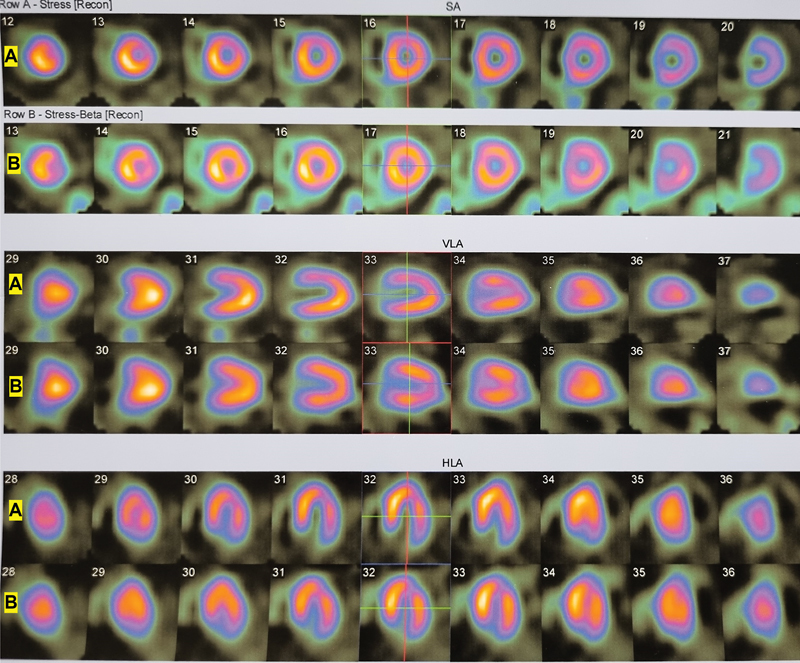
Stress phases of myocardial perfusion SPECT: ( **A** ) with no consumption of beta-blocker which shows decreased perfusion in the anterior and anterolateral segments, and ( **B** ) with consumption of beta-blocker which shows uniform tracer uptake.

The results of our study revealed that most MSPECT indices such as SSS, SDS, TPD, severity, and extension of perfusion defects in RCA, LCX, and LAD territories (based on QPS software polar maps) were significantly reduced after consuming metoprolol compared to the condition in which this medication was discontinued before stress study ( Fig. 1 ). It has been proven that coronary vasodilation following dipyridamole injection is a mechanism for detecting possible myocardial ischemia. 22 23 Since beta-adrenergic receptors bind to similar stimulatory G proteins; thus, inhibitors of those receptors may reduce the activation of responsible G proteins. 24 This inhibitory intervention ultimately leads to a decline in cyclic adenosine monophosphate cell concentration and a decreased response to dipyridamole infusion. 25 We proposed that the effect of beta-blockers by receptor inhibition reduces the sensitivity to dipyridamole and decreases vascular response; however, the determination of the exact mechanism needs more evaluation.

In addition, the only index that did not differ significantly in two conditions of discontinuation and continuation of beta-blockers consumption was the severity reversibility polar map in LAD territory which may be due to the small number of patients who had defects in LAD territory.

Nevertheless, in carvedilol consumers, the results were inconsistent, and we observed that SSS, SDS as well as stress severity score, stress extension score, and TPD in the stress phase in RCA and LCX territories as well as extension reversibility score in RCA territory significantly were higher in the carvedilol consumption phase than in the discontinuation phase. The most important reason is very small sample in the carvedilol group. Carvedilol is not only a beta-adrenoceptor antagonist but also it blocks alpha 1-adrenergic receptors. Probably, this combined action has different effects associated with traditional beta-blockers. 26 27 So, this property may have different effects on the dipyridamole stress and even contribute to the underlying pathway of dipyridamole stress testing. However, considering the very small sample in the carvedilol group and the different involvement of coronary territories between the two studied groups of patients, we cannot comment with certainty on the effect of carvedilol on myocardial perfusion scan. Therefore, to accurately and transparently evaluate the effect of carvedilol, future studies with larger sample sizes and particular carvedilol consumption are needed.

In accordance with our findings, Hoffmeister et al revealed that discontinuation of beta-blockers could increase myocardial blood flow in positron emission tomography (PET) imaging using adenosine. 28 The study of Taillefer et al on 21 patients with proven CAD showed that the use of beta-blocker agents may cause underestimation of the severity of CAD in dipyridamole stress myocardial perfusion scan. In this study, patients were divided into three groups which received placebo, up to 10 mg and up to 20 mg of metoprolol. Taillifer et al showed that SSS and SDS, as well as the size and reversibility of perfusion defects, were significantly reduced in metoprolol receivers compared with placebo ( *p* < 0.05). In this study, the effects of beta-blockers were studied acutely, while our study was performed in patients who used beta-blockers for a long time. 29

Hoffmeister et al examined the effect of beta-blockers on cardiac nuclear imaging in 20 patients with CAD and on long-term treatment with beta-blocker medications, who underwent 13N-ammonia PET with adenosine stress, and then, parametric PET polar maps were converted to virtual SPECT polar maps. The beta-blockers used in this study were metoprolol [25%], nebivolol [20%], carvedilol [5%], and bisoprolol [50%]. In contrast to our findings, they reported no significant difference between the mean score when taking beta-blockers and stopping beta-blockers. 30 In another study by Yoon et al on patients who had coronary angiography in the past 90 days, there was no significant difference between the result of nuclear imaging between the group of patients using beta-blockers and the group that did not use beta-blocker. The mean SSS was 10.4 in the beta-blockers receiver group and 11.1 in the group that did not receive beta-blockers. 31 Table 5 showed summarized results of previous studies about the effect of beta-blocker on myocardial perfusion imaging.

**Table 5:** Results of previous studies about the effect of beta-blocker on myocardial perfusion imaging

Author	Beta-blocker	Paper title	Conclusion or recommendation
Hoffmeister et al 28	No specific	The effect of a beta blocker withdrawal on quantitative adenosine MBF and MPI results.	Most patients: myocardial perfusion imaging results and management decisions are independent of beta-blocker consumption.
Taillefer et al 29	Metoprolol	Effect of acute beta-blockade on dipyridamole Tc-99m Sestamibi myocardial perfusion imaging	Underestimation of the coronary artery disease with consumption of beta-blocker in dipyridamole stress MPI.
Yoon et al 31	No specific	The effect of beta-blockers on the diagnostic accuracy of vasodilator pharmacologic SPECT myocardial perfusion imaging	Beta-blockers: No clinically significant effect on the detection of coronary artery disease.
Fallahi et al 15	No specific	Withholding or continuing beta-blocker treatment before dipyridamole myocardial perfusion imaging for the diagnosis of coronary artery disease	Beta-blocker withholding for diagnostic dipyridamole MPI is not necessary.
Lakkireddy et al 32	No specific	Does beta-blocker therapy affect the diagnostic accuracy of adenosine single-photon-emission computed tomographic myocardial perfusion imaging?	Beta-blocker: No effect on the extent, severity, and reversibility of perfusion defects.
Shehata et al 33	Propranolol	Impact of acute propranolol administration on dobutamine-induced myocardial ischemia as evaluated by myocardial perfusion imaging and echocardiography	Recommended withdrawal of beta-blocker before dobutamine stress.

Abbreviations: MBF, myocardial blood flow; MPI, myocardial perfusion imaging; SPECT, single-photon emission computed tomography.

Dr. Fallahi et al also conducted a randomized clinical trial on 120 patients with proven CAD on coronary angiography with history of treatment with beta-blockers for more than 3 months and investigated the effect of beta-blockers on dipyridamole stress MSPECT. The mean myocardial perfusion scores were not significantly different between the patient in the first group who discontinued beta-blockers before the imaging and the patients in the second group who were not withheld beta-blockers. 15 We suppose that these inconsistencies with our findings may be due to different study designs, the type of beta-blocker, the type of pharmacological stress, studied population, and sample size.

However, the present study was the first within-patient survey to investigate the effect of beta-blockers on myocardial perfusion scans with dipyridamole in Iranian patients who were on long-term treatment with beta-blocker medications. Although this study encountered some limitations such as low sample size, reluctance of patients to participate in the study due to fear of exposure to radiation, and different beta-blockers used by studied patients, there were some valuable strengths. For instance, we compared each patient's MSPECT indices in two conditions of with or without beta-blockers consumption to remove many confounding variables. Also, we selected patients with ischemia to enroll in this study; furthermore, perfusion parameters were evaluated with QPS software, which caused to eliminate operator-dependent errors, and these are the advantages of this study method.

Finally, the results of the present study confirmed that beta-blockers, especially metoprolol, have a significant adverse effect on the diagnostic value of MSPECT scans with dipyridamole stress in terms of underestimating the severity of perfusion defects and decreased diagnostic accuracy of the MSPECT study. So, it is recommended to discontinue beta-blockers before performing an MSPECT procedure with dipyridamole to improve the diagnostic accuracy of the study.

Study limitations: The sample size in our study is relatively small. One of the reasons is ethics for performing two times of stress phase for a person. By the way, we suggest carrying out further multicenter studies with a larger target population and equal medication to obtain more detailed and generalizable results and assess patients with other cardiac medications, especially from alpha- and beta-blocker agents.

## Conclusion

The results of the present study showed that the use of beta-blockers can reduce the imaging accuracy of myocardial perfusion SPECT using dipyridamole. According to these results, discontinuation of beta-blockers, especially metoprolol, 3 days before the scan seems to improve diagnostic accuracy of the dipyridamole MSPECT. On the other hand, other beta-blockers with effect on the other receptors may have different effects.
